# Serum uric acid levels in patients with Familial Mediterranean Fever and healthy controls

**DOI:** 10.1186/1546-0096-13-S1-P83

**Published:** 2015-09-28

**Authors:** B Bitik, S Unverdi, A Tufan, N Yesil, MA Ozturk, M Duranay

**Affiliations:** 1 Ankara Egitim Arastirma Hastanesi, Rheumatology, Ankara, Turkey; 2 Ankara Egitim Arastirma Hastanesi, Nephrology, Ankara, Turkey; 3 Gazi University Faculty of Medicine, Rheumatology, Ankara, Turkey

## Introduction

Familial Mediterranean Fever (FMF) is one of the best described auto-inflammatory diseases. It has also been suggested recently that gout is an autoinflammatory disease. Monosodium urate crystals are known to induce inflammation by complex cellular mechanisms, mainly involving inflammasome and toll-like receptors which are also involved in the pathogenesis of inflammation in FMF. Uric acid itself has been reported to influence inflammatory responses. Hyperuricemia is defined as serum uric acid levels > 6.8 mg/dl. In this study, it was aimed to investigate whether uric acid, which is a well-known risk factor for gout, is also a contributory risk factor for FMF.

## Methods

A retrospective review was made of the charts of a total of 40 patients (23 female, 17 male; mean age: 31± 9.7 years) with FMF and 43 age and gender-matched healthy controls. The patient demographics, clinical findings and serum levels of creatinine, glucose, CRP, uric acid and erythrocyte sedimentation rate were recorded. Patients with creatinine levels > 1.2 mg/dL, renal amyloidosis or diabetes mellitus were excluded from the study.

## Results

The mean serum uric acid levels were 4.5 ± 1.3 mg/dL in patients with FMF and 4.05 ± 1.04 mg/dL in healthy control subjects, and the difference was statistically significant (p = 0.04) [Table 1]. Peritonitis followed by arthritis was the dominant symptom during FMF attacks. Blood tests were applied during an FMF attack in 20 patients. There was no statistically significant difference in respect of serum uric acid between FMF patients with or without an attack (4.3 vs 4.6, respectively, p =0.7). The serum uric acid levels were determined as not significantly different between FMF patients with or without arthritis (4.5 vs 4.4, respectively, p=0.7).

## Conclusion

In this study, serum uric acid levels were found to be higher in FMF patients than in the healthy control subjects. Further prospective studies are needed to reveal the role of uric acid in the pathogenesis of FMF.
